# Prophylactic PDE4 and PDE4B inhibition reduce lesion size and neutrophil infiltration following ischemic stroke in male mice

**DOI:** 10.1177/0271678X251386237

**Published:** 2025-11-03

**Authors:** Laura Ponsaerts, Lotte Alders, Melissa Schepers, Hannelore Kemps, Elke Pirlet, Emily Willems, Mirre De Bondt, Ruben Jacobs, Chiara Brullo, Olga Bruno, Ernesto Fedele, Roberta Ricciarelli, Jos Prickaerts, Veerle Somers, Michiel Vandenbosch, Tim Vanmierlo, Annelies Bronckaers

**Affiliations:** 1 Department of Cardio and Organ Systems, Biomedical Research Institute (BIOMED), Hasselt University, Diepenbeek, Belgium; 2 Division of Translational Neuroscience, Department of Psychiatry and Neuropsychology, Mental Health and Neuroscience Research Institute (MHeNS), Maastricht University, Maastricht, The Netherlands; 3 Department of Neuroscience, Biomedical Research Institute (BIOMED), Hasselt University, Diepenbeek, Belgium; 4 University MS Center (UMSC), Hasselt-Pelt, Belgium; 5 Department of Cardiovascular Sciences, Center for Molecular and Vascular Biology, KU Leuven, Leuven, Belgium; 6 Laboratory of Molecular Immunology, Department of Microbiology, Immunology and Transplantation, Rega Institute for Medical Research, KU Leuven, Leuven, Belgium; 7 BIOMED, Neuro Immune Connections & Repair Lab, Department of Immunology and Infection, Hasselt University, Diepenbeek, Belgium; 8 Division of Imaging Mass Spectrometry (IMS), The Maastricht MultiModal Molecular Imaging (M4I) Institute, Maastricht University, Maastricht, The Netherlands; 9 Section of Medicinal Chemistry, Department of Pharmacy, University of Genova, Genova, Italy; 10 Section of Pharmacology and Toxicology, Department of Pharmacy, University of Genova, Genova, Italy; 11 IRCCS Ospedale Policlinico San Martino, Genova, Italy; 12 Section of General Pathology, Department of Experimental Medicine, University of Genova, Genova, Italy; 13 Peitho Translational, Maastricht, The Netherlands; 14 Department of Immunology and Infection, Biomedical Research Institute (BIOMED), Hasselt University, Diepenbeek, Belgium

**Keywords:** dMCAO, neutrophils, PDE4B, phosphodiesterases, stroke

## Abstract

Stroke remains the second leading cause of death worldwide, highlighting the urgent need for novel treatment options. Phosphodiesterase 4 (PDE4) inhibition has been shown to reduce neuroinflammation and improve neurological outcomes in several neurodegenerative diseases, such as multiple sclerosis. Especially the PDE4B gene is known to contribute to the inflammatory reaction. Therefore, we investigated the effects of PDE4 and PDE4B inhibition in ischemic stroke. We used the distal middle cerebral artery occlusion (dMCAO) mouse model to assess inflammatory cell infiltration and lesion size, and complemented the data with in vitro studies on neutrophils. Our results show that prophylactic PDE4 and PDE4B inhibition reduced the lesion size and neutrophil infiltration in vivo, whereas post-stroke administration of these inhibitors did not show an effect. In vitro, neutrophil activation was decreased following PDE4 and PDE4B inhibition. Furthermore, spatial proteomics analysis of the ischemic brain identified C1QBP as a potential contributing factor to the beneficial effects of prophylactic PDE4B inhibition. Taken together, our research provides evidence for a potential role of prophylactic, but not acute PDE4 and PDE4B inhibition in ischemic stroke treatment, especially for patients at risk of recurrent stroke.

## Introduction

Stroke remains the second leading cause of death worldwide, with 12.2 million new patients suffering a stroke every year. Additionally, stroke accounts for a substantial disability burden in patients, including lasting functional impairments such as gait deficits, language disturbances, and memory deficits.^1–3^ Stroke is classified into two main categories: hemorrhagic stroke and ischemic stroke, the latter accounting for ~87% of all strokes. An ischemic stroke is caused by the occlusion of a major cerebral blood vessel in the brain, whereas hemorrhagic stroke is caused by the rupture of a cerebral blood vessel.^4,5^

The blockage of a major cerebral artery causes an infarct core, which is characterized by irreversible damage, and surrounding salvageable penumbra.^6,7^ Oxygen and nutrient deprivation result in glutamate buildup, triggering calcium influx, oxidative stress, release of reactive oxygen species (ROS) and damage-associated molecular patterns (DAMPs), and excitotoxicity.^5,8–10^ DAMPs initiate a neuroinflammatory cascade, thereby attracting immune cells. Resident microglia are the first responders, producing cytokines and chemokines that recruit peripheral immune cells. Neutrophils are the earliest peripheral responders, followed by monocytes and macrophages, peaking within 3–7 days post-stroke. This acute immune phase exacerbates brain damage, while the chronic phase involves T and B cells, prolonging inflammation, and hindering recovery.^8,11–13^ The pro-inflammatory reactions exerted by peripheral immune cells contribute further to brain tissue damage and subsequent functional impairment following ischemic stroke.

Current treatment options for ischemic stroke include recombinant tissue plasminogen activators (rtPAs) and mechanical thrombectomy.^14–17^ Both aim to restore cerebral blood flow by dissolving or physically removing the blockage. However, only a limited number of patients can benefit from these therapies due to several contraindications (e.g. hypertension, history of stroke) and limited therapeutic time window.^14–17^ Therefore, there remains an urgent medical need for improved treatment options for ischemic stroke. Additionally, patients who have already suffered a stroke are at risk of stroke recurrence. For these patients, the prevention strategy still largely consists of antithrombotic medication and changes in their lifestyle.^18–20^ This particular subset of patients might benefit from a prophylactic treatment approach to reduce neuroinflammation and lesion size following a secondary stroke.^21,22^

A potential novel treatment strategy could consist of selective phosphodiesterase (PDE) inhibition. Cyclic nucleotide phosphodiesterases are a group of enzymes that hydrolyze cyclic adenosine monophosphate (cAMP) and cyclic guanosine monophosphate (cGMP).^23–26^ Both cAMP and cGMP are second messengers that play key roles in cell signaling functions. PDEs are classified into 11 families (PDE1–11), which are further divided into different genes (e.g. PDE4A–D). Each PDE gene contains several different isoforms (e.g. PDE4B1–PDE4B5) due to alternative splicing and the use of alternative promoters.^25–27^ PDE families show a distinct expression pattern across tissues and cell types. Among these, the PDE4 family is the highest expressed cAMP-specific PDE family in brain tissue and inflammatory cells.^25,27^

Previous preclinical research has shown beneficial effects of PDE4 inhibition following experimental ischemic stroke in rodents. Kraft et al. studied the effects of PDE4 inhibition using the first-generation pan-PDE4 inhibitor rolipram in mice following transient middle cerebral artery occlusion (tMCAO). Rolipram reduced the lesion size while improving neurological scores 24 h following stroke induction. Additionally, rolipram reduced neuroinflammation and rescued blood–brain barrier (BBB) damage.^28^ A second-generation PDE4 inhibitor, roflumilast, also reduced neuroinflammation and improved cognition in mice undergoing bilateral common carotid artery occlusion.^29^ A variety of pan-PDE4 inhibitors have shown similar results in rat ischemic stroke models, including reduced lesion sizes, improved neurological scores, decreased neuronal apoptosis, reduced BBB disruption, and reduced neuroinflammatory effects.^30–34^ However, despite the beneficial neuroprotective and anti-inflammatory effects of pan-PDE4 inhibitors in experimental stroke models, their clinical use is hindered by severe emetic side effects.^35,36^ Because of this major limitation, our study investigates the effect of more specific PDE4B inhibition on lesion size and the neuroinflammatory reaction following experimental ischemic stroke in mice. We hypothesized that, similar to pan-PDE4 inhibition, selective PDE4B inhibition exerts anti-inflammatory effects without triggering emetic side effects.^37^

Indeed, PDE4B inhibition has been shown to be beneficial in traumatic brain injury (TBI) and experimental autoimmune encephalomyelitis, both of which are characterized by neuroinflammation.^38–40^ In rat models of TBI, PDE4B inhibition resulted in reduced inflammation, decreased neuronal impairment, and improved memory performance.^38,39^ Wilson et al. reported reduced neutrophil infiltration into the affected brain area upon PDE4B inhibition 24 h following experimental TBI.^38^ Furthermore, PDE4B inhibition using A33 also leads to reduced levels of tumor necrosis factor-α (TNF-α) post-TBI.^39^ These positive results contributed to our hypothesis, stating that PDE4B inhibition dampens neuroinflammation and reduces the lesion size in a distal middle cerebral artery occlusion (dMCAO) mouse model for ischemic stroke. Additionally, polymorphisms of the PDE4D gene have been linked to ischemic stroke susceptibility.^41–43^ PDE4D inhibition has been shown to stimulate remyelination in a mouse model for multiple sclerosis, while also improving functional outcome following experimental spinal cord injury.^40,44^ Therefore, we hypothesized that also PDE4D inhibition could exert a neuroprotective effect following ischemic stroke. To test our hypotheses, we compared the effects of both prophylactic and acute administration of pan-PDE4 inhibition (roflumilast) to PDE4B (A33) and PDE4D (Gebr32a) inhibition on the neuroinflammatory reaction following ischemic stroke using flow cytometry. Furthermore, we assessed the pan-PDE4, PDE4B, and PDE4D inhibition effect on the lesion size at different time points following dMCAO stroke by means of 2,3,5-triphenyltetrazolium chloride (TTC) staining. Finally, we employed a spatial proteomics approach to identify proteins potentially contributing to the beneficial effects of prophylactic PDE4B inhibition following stroke.

## Materials and methods

### Animals

Nine-week-old male C57BL/6 were purchased from Envigo (Horst, The Netherlands). Mice were housed in compliance with European guidelines for animal experimentation, on a 12-h light–12-h dark cycle with access to food and water ad libitum. All animal experiments were approved by the ethical committee for animal experimentation at Hasselt University (ethical matrices ID 202062, ID 202236), and in accordance with the EU directive 2010/63/EU and the Belgian law of animal welfare and Royal Decree of the May 29, 2013, and the ARRIVE guidelines.

### Distal middle cerebral artery occlusion (dMCAO) stroke surgery

Experimental ischemic stroke was induced in 10-week old male C57Bl/6 mice using the permanent dMCAO model as previously described.^45^ In short, mice were anesthetized using 2% isoflurane and an incision was made between the left ear and eye, after which the temporal muscle was dissected to expose the bifurcation of the middle cerebral artery (MCA). Next, the skull above this bifurcation was carefully thinned and removed using a microdrill. The MCA was coagulated proximal of the bifurcation using electrocoagulation. To protect the eyes from drying out during surgery, eye ointment was applied. During surgery, mouse body temperature was maintained using a heating pad. Following surgery, mice were allowed to regain consciousness in a heating cage (35 °C). Additionally, mice received 0.05 mg/ml buprenorphine analgesic by subcutaneous injection for 3 days post-surgery. Animals that died during or after surgery were excluded from the study.

### PDE4, PDE4B, and PDE4D inhibition treatment

For all experiments, mice were randomly divided into different treatment groups. Mice received their respective treatment by subcutaneous injection in a prophylactic manner prior to stroke induction, or acutely following stroke induction. Treatment groups included DMSO (0.1% or 0.3%, vehicle control), and different concentrations (3 and 10 mg/kg) of roflumilast (pan-PDE4 inhibitor, IC50 = 0.2–4.3 nM^46^; Leader Biochemical Group), A33 (PDE4B inhibitor, IC50 = 27 nM^47^; Sigma–Aldrich), and Gebr32a (PDE4D inhibitor, IC50 = 1.16–4.97 µM^48^; University of Genova).^48^ Treatment injections further contained 2% Tween80 (Merck, NJ, USA) in 0.5% methylcellulose (Sigma–Aldrich, MO, USA). Dosing of 3 mg/kg of roflumilast and A33 was based on positive results of previous research in animal models for multiple sclerosis and spinal cord injury.^40,44^ Dosing of Gebr32a was equated to that of roflumilast and A33 since it was shown not to induce emetic side effects in the ketamine/xylazine test.^48^ Mice were treated prophylactically, 24 h prior to dMCAO stroke, or acutely following stroke induction. Animals were sacrificed either 24 h or 7 days after stroke and brains were analyzed using TTC staining or flow cytometry. Further details on the different in vivo experiments carried out in this study are shown in Supplementary Table 1.

### Tissue sample preparation—TTC staining—Flow cytometry

For the assessment of lesion size, mice were sacrificed using cervical dislocation. Whole brains were isolated and brain slices of 1 mm were made and stained in a 2% TTC solution (Sigma–Aldrich) for at least 30 min. Stained brain slices were digitally photographed and lesion size was quantified using ImageJ Software (NIH, Bethesda, USA). Percentage lesion was calculated as total lesion volume/total brain volume, multiplied by 100. The researcher who performed the analysis was a different research from the one that injected the mice, and the analysis was performed blinded.

For the assessment of neuroinflammation, mice were sacrificed using transcardial perfusion. Mice were anesthetized using an intraperitoneal injection of Dolethal (200 mg/kg; Vetoquinol, Niel, Belgium). Following perfusion with heparin in saline solution, brains were isolated and divided into ipsilateral and contralateral hemispheres. Next, brain hemispheres were mechanically dissociated and passed once through a 70 µm tissue filter to create a cell suspension. Next, cells were stained with fluorescently labeled antibodies CD11b, CD45, CD11c, Ly6C, Ly6G, CCR2, and Arginase (Supplementary Table 2) for 15 min. Stained cell suspensions were analyzed using the Fortessa flow cytometer (BD LSRF Fortessa; BD Biosciences, NJ, USA; Supplementary Figure 3). Neutrophils were identified as cells positive for CD45, CD11b, and Ly6G^high^. Macrophages were identified as cells positive for CD45, CD11b, Ly6C^high^, and Arginase. Monocytes were identified as cells positive for CD45, CD11c, Ly6G^low^.

### Cylinder test

For the assessment of functional recovery following dMCAO stroke, the cylinder test was employed as previously described.^49,50^ In short, two mirrors were placed at an angle of 45° behind a plastic see-through cylinder, and a digital camera was placed in front of the cylinder so that all sides of the cylinder were visible. When a mouse is placed inside of the cylinder, its natural behavior is to start exploring the walls by standing on its rear legs and touching the sides with its front paws. Contralateral and ipsilateral paw touches were quantified. Following ischemic stroke, mice will show a preference to using their ipsilateral paw. Analysis of the impaired forelimb use was done as followed: $\begin{array}{l} \%\;\;\mathrm{impaired}\;\mathrm{forelimb}\;\mathrm{use}=\\ \frac{\mathrm{number}\;\;\mathrm{of}\;\;\mathrm{contralateral}\;\;\mathrm{paw}\;\;\mathrm{touches}}{\mathrm{total}\;\mathrm{number}\;\mathrm{of}\;\;\mathrm{paw}\;\;\mathrm{touches}}\times 100 \end{array}$ , and normalization was done against baseline values by dividing the % impaired forelimb use by the % impaired forelimb use at baseline. The cylinder test was performed for each mouse at baseline, meaning 1 day before dMCAO stroke induction; and again on days 1, 3, 7, and 14 post-stroke.

### Neutrophil chemiluminescence assay

Primary human neutrophils were immunomagnetically isolated out of whole blood samples, using the EasySep human neutrophil isolation kit (StemCell Technologies, catalog number 19666, Vancouver, Canada) according to manufacturer’s instructions. Whole blood samples were collected from healthy donors who provided informed consent, under approval from the University Biobank Limburg (UbiLim, Hasselt, Belgium, ethical matrix CME2022/020) and in accordance with the ethical principles as stated in the latest version of the Declaration of Helsinki, Good Clinical Practice, and the Belgian law of May 7, 2004. Isolated neutrophils (150,000 cells/well of a 96-well plate) were treated with DMSO (0.1%), roflumilast (0.1 and 1 µM), or A33 (0.03, 0.1, and 1 µM) prophylactically (1 h prior to inflammatory stimulus), acutely (simultaneous with inflammatory stimulation), or 15 min post-inflammatory stimulation. The inflammatory stimulus was a combination of TNF-α (50 ng/ml, 300-01A; Peprotech, NJ, USA) and IL-1β (500 ng/ml, 200-01B; Peprotech, NJ, USA). Luminol (5 mM; Sigma–Aldrich, MO, USA) was added and converted by ROS produced by activated neutrophils into a chemiluminescent signal, which was quantified using the ClarioStar plus plate reader (BMG Labtech, Ortenberg, Germany). Data were normalized against the DMSO control per experiment.

### qRT-PCR analysis of PDE4B isoforms

Primary neutrophils (isolated as described above) were left unstimulated. After incubation at 37 °C for 2 h, cells were centrifuged and the pellet was resuspended in Qiazol (Qiagen, Venlo, The Netherlands). RNA was extracted from samples using standard chloroform RNA extraction. RNA concentration was determined using the Nanodrop 2000 system (Thermo Scientific, MA, USA), and 180 ng/µL of RNA was converted into cDNA using QScript (4 µL, Quantabio, MA, USA). qPCR was performed on the sample of cDNA (22.5 ng) to determine the PDE4B isoforms in a mixture with Sybrgreen (Thermo Fisher, MA, USA), MQ, and corresponding primers (10 µM, Supplementary Table 3, IDT, IA, USA). *Ct* values obtained from qPCR analysis using Quantstudio 3 (Applied Biosystems, CA, USA) were calculated into startfluorescence. Startfluorescence was calculated using following formula: *Ct* threshold − (log slope × *Ct* value sample), this result was transformed by using 10^result^. The log slope value was determined using primer efficiency.

### LC–MS proteomics

#### Laser microdissection

For this experiment, mice were operated as described above. Treatment groups included DMSO (0.1%), roflumilast prophylactic (3 mg/kg), and A33 prophylactic (3 mg/kg) with a sample size of four mice per group. Twenty-four hours post-stroke, mice were sacrificed by means of cervical dislocation and brains were isolated and cryofrozen in liquid nitrogen to store at −80 °C. Cryosections of 10 µm thickness were made, and for each mouse the stroke lesion was identified using cresyl violet (0.1%; Sigma–Aldrich).^51^ Annotations of the regions of interest, namely the penumbra were made using QuPath-0.5.1 following digitalization of the brain sections using the axioscan Z1 (Zeiss, Oberkochen, Germany; Supplementary Figure 4). Laser microdissection of these annotated areas was performed with a Leica LMD 7000 (Leica Microsystems) using the “draw and cut” method. One square millimeter regions were cut out and collected in 0.5 ml tubed containing 20 µl ammonium bicarbonate buffer 50 mM (ABC). Samples were stored at −80 °C until sample preparation for LC–MS analysis.

#### Sample preparation for proteomics

Samples were processed following the protocol described by Mezger et al.^52^ Briefly, Rapigest was added to a final concentration of 0.1% and samples were incubated in a Thermoshaker (Eppendorf, Hamburg, Germany) at 800 rpm at room temperature (RT, 20 °C). Reduction was carried out by adding dithiothreitol (DTT, final concentration 10 mM), followed by incubation at 56 °C for 40 min at 800 rpm. Alkylation was then performed using iodoacetamide (IAM, final concentration 20 mM) at RT for 30 min, under continuous shaking. To quench access IAM, DTT was re-added (final concentration 10 mM) and incubated at RT for an additional 10 min. Proteolytic digestion was carried out in two steps. First trypsin/Lys-C (15 µg/ml), dissolved in resuspension buffer (50 mM acetic acid, pH <3), was added and samples were incubated overnight at 37 °C under shaking. A second digestion was performed by adding 0.3 µl trypsin (final concentration 5 µg/ml) in 80% acetonitrile (ACN), followed by incubation at 37 °C for 3 h. Digestion was terminated with trifluoroacetic acid (TFA, final concentration 0.5%) and incubated at 37 °C for 45 min. Samples were centrifuged at 15,000*g* for 10 min at 4 °C and the supernatant was collected and concentrated using a SpeedVac concentrator (Hetovac VR-1; Heto Lab Equipment, Denmark) to a final volume of 50 µl. Processed samples were stored at −80 °C until LC-MS analysis.

#### Liquid chromatography–mass spectrometry (LC–MS)

A Thermo Scientific (Dionex) Ultimate 3000 Rapid Separation UHPLC system equipped with a Thermo Scientific Acclaim PepMap C18 analytical column (15 cm, ID 75 and 3 µM) was used to perform peptide separation. The mobile phase consisted of 0.1% FA in UPLC-grade water (eluent A) and 0.1% FA in 20:80 water/acetonitrile (ACN; eluent B), at a 300 nL/min flow rate. The peptide samples were desalted on an online installed C18 trapping column. Next, peptides were separated on the analytical column using a stepwise gradient of 5%–27.5% eluent B for 28 min, followed by an increase of 27.5%–40% eluent B from 28 to 30 min, 40%–90% eluent B from 30 to 31 min, and finished with a washing step at 95% eluent B for 4 min. Next, the gradient was restored to 96% eluent A, 5% eluent B for 20 min. The UHPLC system was coupled to a Q Exactive HF mass spectrometer (Thermo Scientific), and the mass spectrometry was operated in Data Independent Acquisition (DIA) mode. Settings were adapted from Pino et al.^53^ In short, the Thermo Q-Exactive HF was configured to acquire 25 × 24 m/z (400–1000 m/z) precursor isolation window DIA spectra at resolution of 30,000 using a staggered window pattern. Precursor spectra, mass range 385–1015 m/z at 60,000, were interspersed every 25 MS/MS spectra. For protein identification and quantification, the DIA spectra were analyzed using DIA-NN version 1.8.1.^54^ The DIA-NN software was operated in library-free mode using the Swissprot *Mus musculus* protein database (*Mus musculus*, TaxID = 10090). Cysteine carbamidomethylation and N-terminal methionine excision were set as fixed modifications. Label-free quantification was performed in DIA-NN using MaxLFQ and RT-dependent cross-run normalization.^55^ Following data analysis of significant up- and down-regulated proteins was performed using GraphPad Prism 9.2.0 software.

### Immunohistochemistry staining

Brain slices of mice used in the proteomics experiment were also used for immunohistochemistry staining. The cryosectioned brain slices were permeabilized using 0.05% Triton X-100 and protein blocked using 10% Dako protein block (Dako, Glostrup, Denmark). Next, brain slices were incubated with primary antibodies for 2 h at RT. Primary antibodies included rabbit anti-C1QBP (1/500 dilution, catalog number 24474-1-AP, Proteintech, IL, USA) and mouse anti-NeuN (1/500 dilution, catalog number MAB377; EMD Millipore, MA, USA). Following incubation, brain slices were washed and incubated for 1 h at RT using the secondary antibodies donkey anti-rabbit (Alexa fluor 488, 1/500 dilution, catalog number A32790; Invitrogen) and donkey anti-mouse (Alexa fluor 555, 1/500 dilution, catalog number A31570; Invitrogen). Then, brain slices were washed again and incubated with 0.1% Sudan black for 30 min at room temperature. Brain slices were stained using DAPI (1/10,000 dilution; Thermo Scientific) and mounted using fluoromount (Invitrogen). Stained brain slices were scanned using the axioscan Z1 and analyzed for fluorescent intensity using the Zeiss Zen lite 3.11 software (Zeiss). Additionally, images of the brain slices were obtained using a fluorescence microscope (Leica DM4000 B LED, Wetzlar, Germany). Fluorescent intensity was statistically analyzed using GraphPad Prism 9.2.0 software.

### Statistical analyses

Statistical analysis was performed using GraphPad Prism 9.2.0 software (La Jolla, CA, USA). All data were checked for normality and normal distribution using the Shapiro–Wilk test. When data passed the normality test, a parametric statistical test was used. When data did not pass normality testing, a non-parametric statistical analysis was performed. In general, when two groups were included in the analysis, a *t*-test or Mann–Whitney *U*-test was performed. When more than two groups were included, a one-way ANOVA or Kruskal–Wallis analysis was employed with Tukey correction for multiple comparisons. All data are represented as mean ± standard deviation (SD).

## Results

### Prophylactic PDE4 and PDE4B inhibition reduce lesion size following dMCAO stroke induction

The in vivo treatment effects of PDE4 (roflumilast), PDE4B (A33), and PDE4D (Gebr32a) inhibition following dMCAO were investigated using two different treatment regimes. Mice either received the inhibitor prophylactically, 24 h prior to stroke induction, or acutely starting 2 h post-stroke induction (Figure 1(a) and (d)). The effect on lesion size was assessed 24 h following experimental ischemic stroke using a TTC staining (Figure 1(b) and (e)). Prophylactic treatment with roflumilast (3.83% ± 1.91%) or A33 (3.27% ± 1.92%), but not Gebr32a, significantly reduced the lesion size, respectively, by 49.8% (*p* = 0.031) and 57.2% (*p* = 0.0056) compared to DMSO treatment (7.63% ± 2.99%; Figure 1(c)). However, acute PDE4 and PDE4B inhibition treatment did not affect lesion size compared to the DMSO control (Figure 1(f)). Additionally, PDE4D inhibition did not show any effect on the lesion following prophylactic and acute treatment (Figure 1(c) and (f)).

**Figure 1.**
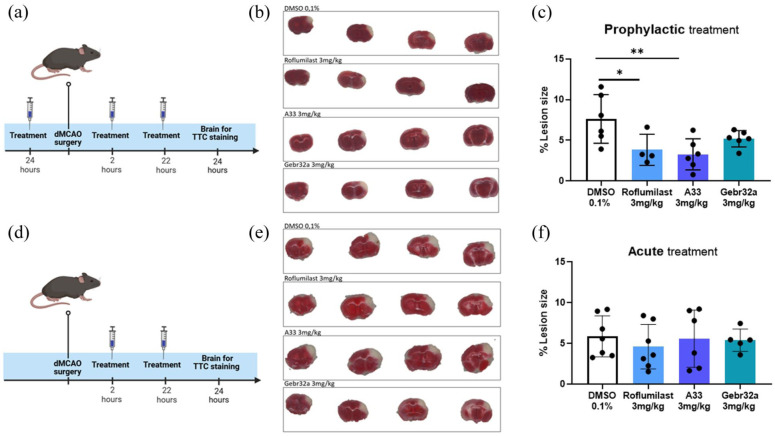
Prophylactic, but not acute, PDE4 and PDE4B inhibition reduces lesion size in a dMCAO mouse model for ischemic stroke. (a) Timeline of the prophylactic treatment experiment. C57Bl/6 mice received their first subcutaneous treatment injection 24 h prior to dMCAO surgery. The treatment injection was repeated 2 and 22 h after dMCAO surgery. Twenty-four hours post-stroke induction, brains were isolated for TTC staining. Treatment groups included: DMSO (0.1%, vehicle control), roflumilast (3 mg/kg, PDE4 inhibitor), A33 (3 mg/kg, PDE4B inhibitor), and Gebr32a (3 mg/kg, PDE4D inhibitor). Created with BioRender.com (b) representative TTC images. (c) Quantification of lesion size assessed with TTC staining. Both roflumilast and A33 showed a reduction in lesion size compared to DMSO (*n* = 4–6/group). (d) Timeline of the acute treatment regime. Stroke was induced using the dMCAO technique. Mice received their subcutaneous treatment injection 2 and 22 h following dMCAO surgery. Twenty-four hours post-stroke induction, brains were isolated for TTC staining. Treatment groups included: DMSO (0.1%), roflumilast (3 mg/kg), A33 (3 mg/kg), and Gebr32a (3 mg/kg). Created with BioRender.com (e) representative TTC images. (f) Quantification of TTC stainings. No significant differences were observed in lesion size following acute treatment (*n* = 5–7/group). Data are represented as mean ± SD. Statistical analysis included one-way ANOVA analysis with a Tukey correction for multiple comparisons. dMCAO: distal middle cerebral artery occlusion; TTC: 2,3,5-triphenyltetrazolium chloride. *p < 0.05. **p < 0.005.

To further investigate a potential effect on lesion size following acute PDE4 and PDE4B inhibition, mice received a higher dosage of roflumilast at 10 mg/kg based on previous research by Kraft et al.^28^ However, roflumilast at 10 mg/kg did not show a significant effect on lesion size compared to DMSO treated mice (Supplementary Figure 1(a)–(c)). Taking the half-life of the inhibitors into account, we studied the effect of roflumilast at different doses (i.e. 3 and 10 mg/kg) on lesion size development, where mice received their treatment injections every 8 h during a 24-h period. No significant effect of roflumilast at 3 or 10 mg/kg was observed compared to DMSO (Supplementary Figure 1(d)–(f)). Next, the effect of prolonged acute PDE4 and PDE4B inhibition was assessed. Mice received their respective treatment at a dose of 3 mg/kg twice/day for a period of 7 days following stroke induction. Seven days post-stroke, TTC staining showed no significant lesion size reduction with neither roflumilast nor A33 compared to DMSO (Supplementary Figure 1(g)–(i)). Additionally, we studied the effect of inhibition treatment on sensorimotor function in the dMCAO stroke model employing the cylinder test (Supplementary Figure 2(a)). Acute treatment with roflumilast, A33, or Gebr32a did not show any significant effects on the impaired forelimb use compared to the DMSO control at day 1, 3, 7, or 14 following dMCAO stroke induction (Supplementary Figure 2(c)–(f)).

### Prophylactic PDE4 and PDE4B inhibition reduce neutrophil infiltration following experimental stroke in mice

Since high PDE4 and PDE4B expression has previously been reported in inflammatory cells,^37^ a potential effect of the PDE4 and PDE4B inhibitors on peripheral immune cell infiltration following experimental stroke was investigated by means of flow cytometry analysis (Figure 2(a) and (e)). A significant reduction in the relative percentage of infiltrated neutrophils was observed upon prophylactic treatment with roflumilast (0.45% ± 0.25%, *p* = 0.028) and A33 (0.46% ± 0.20%, *p* = 0.025) compared to DMSO treated mice (1.00% ± 0.30%; Figure 2(b)). However, this significant reduction in infiltrated neutrophils was not discovered in acutely-treated mice (Figure 2(f)). Additionally, prophylactic and acute PDE4D inhibition using Gebr32a did not yield any significant effect on infiltrated neutrophils post-stroke (Figure 2(b) and (f)). Interestingly, prophylactic treatment with the PDE4B inhibitor A33 (5.33% ± 3.53%, *p* = 0.0025) also significantly increased the relative percentage of arginase positive macrophages compared to DMSO (1.00% ± 0.15%; Figure 2(d)). This effect was not observed after prophylactic or acute PDE4B inhibition on the relative percentage of infiltrated arginase positive monocytes compared to DMSO (Figure 2(c) and (g)).

**Figure 2.**
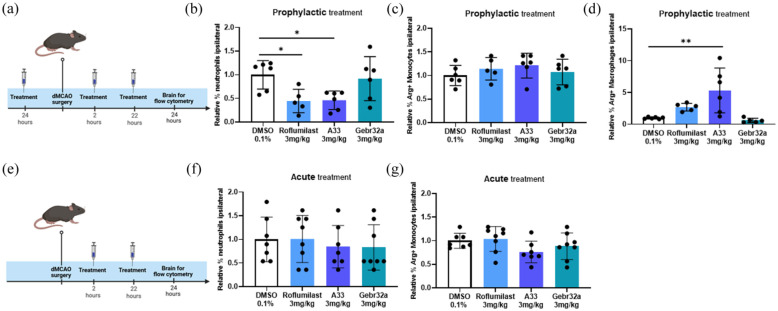
Prophylactic, but not acute, PDE4 and PDE4B inhibition reduce neutrophil infiltration in a dMCAO mouse model for ischemic stroke. (a) Timeline of the experiment. Mice received a first subcutaneous treatment injection 24 h prior to dMCAO surgery, and received another treatment injection 2 and 22 h post-stroke induction. Twenty-four hours following dMCAO stroke, brains were isolated and processed for flow cytometry analysis. Treatment groups included: DMSO (0.1%, vehicle control), roflumilast (3 mg/kg, PDE4 inhibitor), A33 (3 mg/kg, PDE4B inhibitor), and Gebr32a (3 mg/kg, PDE4D inhibitor). Created with BioRender.com (b) quantification of the relative percentage infiltrated neutrophils in the ipsilateral hemisphere (*n* = 5–6/group). (c) Quantification of the relative percentage infiltrated monocytes in the ipsilateral hemisphere (*n* = 5–6/group). (d) Quantification of the relative percentage infiltrated macrophages in the ipsilateral hemisphere (*n* = 5–6/group). (e) Timeline of the experiment. Mice received a treatment injection 2 and 22 h post-stroke. Twenty-four hours following dMCAO stroke, brains were isolated and processed for flow cytometry analysis. Treatment groups included: DMSO (0.1%), roflumilast (3 mg/kg), A33 (3 mg/kg), and Gebr32a (3 mg/kg). Created with BioRender.com (f) quantification of the relative percentage infiltrated neutrophils in the ipsilateral hemisphere (*n* = 7–8/group). (g) Quantification of the relative percentage infiltrated monocytes in the ipsilateral hemisphere (*n* = 7–8/group). Data are represented as mean ± SD. Statistical analysis included one-way ANOVA analysis with a Tukey correction for multiple comparisons. dMCAO: distal middle cerebral artery occlusion. *p < 0.05. **p < 0.005.

### Roflumilast and A33 reduce the production of reactive oxygen species in neutrophils in vitro

Considering that prophylactic treatment with roflumilast and A33 significantly reduced the percentage of brain-infiltrated neutrophils in the dMCAO stroke model, we investigated the effect of these inhibitors on neutrophil activation in vitro. Primary human neutrophils were isolated from fresh whole blood samples using immunomagnetic isolation. These isolated neutrophils were treated either prophylactically, acutely, or 15 min post-inflammation with different concentrations of roflumilast (0.1 or 1 µM) or A33 (0.03, 0.1, or 1 µM). ROS produced by inflammatory activated neutrophils was quantified using chemiluminescence (Figure 3(a)). Roflumilast at 1 µM significantly lowered neutrophil activation compared to the DMSO control when administered prophylactically (0.24 ± 0.26, *p* < 0.0001), acutely (0.53 ± 0.19, *p* = 0.0005), and 15 min post-inflammation (0.53 ± 0.24, *p* = 0.0045; Figure 3(b)–(d)). A33 at 0.1 µM significantly reduced neutrophil activation when administered prophylactically (0.67 ± 0.24, *p* = 0.0362) and acutely (0.59 ± 0.19, *p* = 0.0085), but not when administered 15 min post-inflammation (1.12 ± 0.19; Figure 3(e)–(g)). Additionally, roflumilast at 0.1 µM and A33 at 0.03 and 1 µM did not significantly affect neutrophil activation, suggesting a dose-dependent effect of both inhibitors on ROS production, following neutrophil activation.

**Figure 3.**
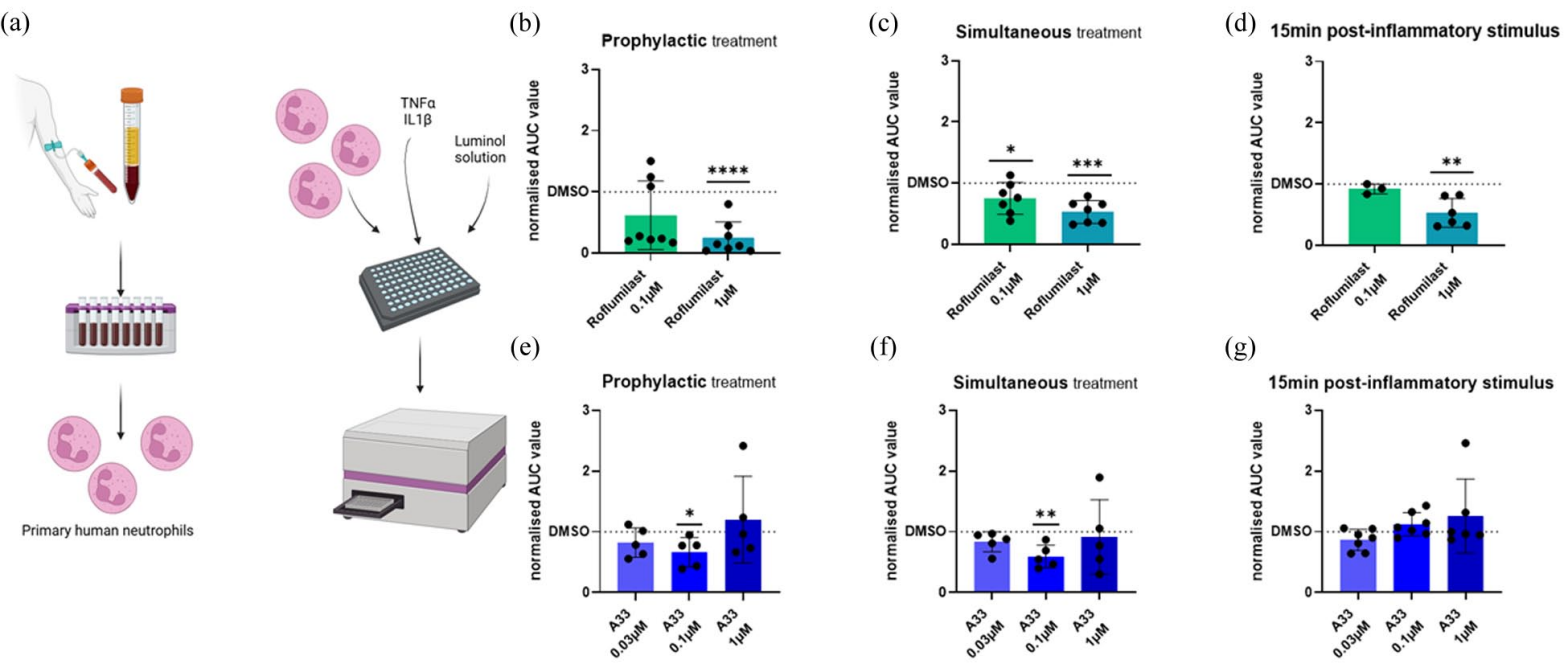
PDE4 and PDE4B inhibition reduce the production of reactive oxygen species by human neutrophils. (a) Schematic overview of the experiment. Neutrophils were isolated from human whole blood. Next, neutrophils were treated with DMSO (0.1%), roflumilast (PDE4 inhibitor, 0.1 or 1 µM), or A33 (PDE4B inhibitor, 0.03, 0.1, or 1 µM) either prophylactically (1 h prior to inflammatory stimulus), acutely (simultaneously with inflammatory stimulus), or 15 min post-inflammatory stimulus. Inflammatory stimulation was evoked by a combination of TNF-α (50 ng/ml) and IL-1β (500 ng/ml). Activated neutrophils produce ROS that convert luminol into a chemiluminescent signal that was measured using a Clariostar plate reader. Created with BioRender.com (b–g) AUC values of ROS production over time (3 h), and normalized to the AUC value of the DMSO control (0.1%). Cells were treated with (b–d) roflumilast (0.1 or 1 µM) or (e–g) A33 (0.03, 0.1, or 1 µM). Cells were treated (b, e) before inflammatory stimulus (prophylactic) or (c, f) simultaneous with inflammatory stimulus or (d, g) 15 min after inflammatory stimulus. Data are represented as mean ± SD and were normalized against the DMSO control per experiment. Statistical analysis one sample *t*-test was performed. ROS: reactive oxygen species. *p < 0.05. **p < 0.005. ***p < 0.0005. ****p < 0.0001.

### PDE4B5 and PDE4B2 are the main expressed PDE4B isoforms in human neutrophils

Next, we determined which PDE4B isoforms are mainly expressed in primary human neutrophils using qRT-PCR analysis (Figure 4(a)). We found that mainly PDE4B5 and PDE4B2 were expressed in neutrophils that have not been activated by an inflammatory stimulus. In fact, PDE4B5 (8.738 ± 9.071e−011, *p* < 0.0001) was significantly more expressed than PDE4B3 (2.002 ± 4.528e−017), and PDE4B2 (5.294 ± 7.091e−014; Figure 4(b)). PDE4B1 was not detected in these samples.

**Figure 4.**
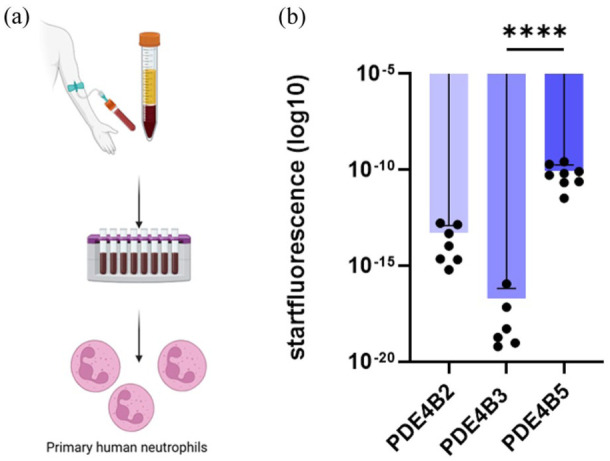
PDE4B isoform expression in unstimulated primary human neutrophils. (a) Neutrophils were isolated from human whole blood using immunomagnetic isolation. Next, neutrophils were left unstimulated and incubated at 37 °C for 2 h. RNA was collected from these neutrophils and expression of different PDE4B isoforms was analyzed using qRT-PCR. Created with BioRender.com (b) startfluorescence in a Log10 scale of PDE4B2, PDE4B3, and PDE4B5. Data are represented as mean ± SD. Statistical analysis Kruskal–Wallis test was performed using GraphPad Prism. ****p < 0.0001.

### Spatial proteomics analysis reveals differences in C1QBP expression after PDE4 and PDE4B inhibition in mice following ischemic stroke

To further unravel the underlying mechanisms of prophylactic PDE4 and PDE4B inhibition following ischemic stroke in mice, we determined the effect of inhibition treatment on protein expression in the brain using a spatial proteomics analysis. We included proteins that were found in at least three samples for each group, and zero values were imputed by low values. *T*-tests were performed between groups and significant proteins were identified using nominal *p* values (*p* < 0.05). Mice treated with DMSO were compared to mice prophylactically treated with roflumilast, and 100 significantly up- and downregulated proteins were identified (Figure 5(b)). The three most downregulated proteins for this comparison included nuclear receptor coactivator 5 (NCOA5), myosin light chain 3 (MYL3), and linker histone cluster member H14/H15. The three most upregulated proteins were importin 9 (IPO9), coenzyme 9 (COQ9), and microtubule associated protein (MARE1, MARE3). We also compared mice treated with DMSO to mice prophylactically treated with A33 and identified 74 significantly up- and downregulated proteins (Figure 5(c)). For this comparison, the three most downregulated proteins included serpine mRNA binding 1 (SERB1), myosin light chain 3 (MYL3), and NADH-ubiquinone oxidoreductase chain 1 (NU1M), while the three most upregulated proteins were identified as S100 calcium binding protein 1 (S100A1), non-specific serine/threonine protein kinase (KC1E), and nuclear receptor binding protein 2 (NRBP2). However, none of these most up- and downregulated proteins were reported to play a role in the neuroinflammatory process, which is of interest following stroke. Therefore, we performed a hypothesis-driven ontology analysis using the online tool GOrilla for both comparisons for biological process and molecular function. This analysis revealed a limited ontology effect for the proteins identified in the comparison of DMSO treatment to prophylactic roflumilast treatment (Figure 5(f)). However, this was more extensive for the comparison of DMSO treatment to prophylactic A33 treatment, which revealed amongst others an effect on the immune response (Figure 5(g)). One of the proteins that contributed to this immune response effect was the complement C1q binding protein (C1QBP) which we ranked first based on involvement in the amount of biological processes and molecular functions. Additionally, we found that C1QBP is significantly downregulated (nominal *p* = 0.023) following prophylactic treatment with A33 compared to DMSO in the ischemic brain (Figure 5(e)). Interestingly, C1QBP was upregulated following prophylactic roflumilast treatment compared to DMSO (Figure 5(d)).

**Figure 5.**
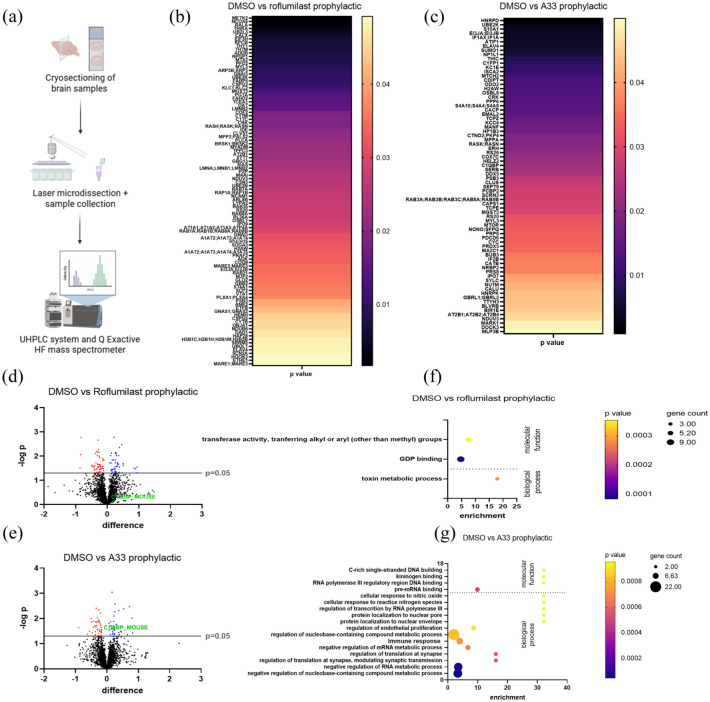
Proteomics analysis results following prophylactic PDE4 and PDE4B inhibition in an ischemic stroke mouse model. (a) Schematic overview of the experiment. Laser microdissection was performed on the region of interest on 10 µm brain sections, and samples were collected for sample preparation. Proteins were identified using the UHPLC system and Q exactive HF mass spectrometer. Heat map showing significantly up- and downregulated proteins (nominal *p* < 0.05) for mice treated with DMSO compared to roflumilast prophylactic treatment. Created with BioRender.com (b) and DMSO compared to A33 prophylactic treatment (c). Volcano plot showing all identified proteins and significantly up (blue)- and downregulated (red) proteins for DMSO treatment compared to roflumilast prophylactic treatment (d) and DMSO treatment compared to A33 prophylactic treatment (e). Bubble plots showing protein ontology analysis for DMSO treatment compared to roflumilast prophylactic treatment (f) and DMSO treatment compared to A33 prophylactic treatment (g). Data analysis was performed using Perseus software. Statistical analysis of Volcano plot and Bubble plot was performed using GraphPad Prism software.

Because of the link of C1QBP to several biological processes and especially to immune response, C1QBP can be an interesting target following PDE4B inhibition to potentially lower neuroinflammation following stroke. Therefore, we performed immunofluorescent staining to visualize this protein in brain sections. We confirmed that prophylactic A33 treatment showed a trend towards lower C1QBP expression compared to DMSO treated mice in the penumbra (Figure 6(e)). Additionally, prophylactic roflumilast treatment showed a trend towards increased C1QBP expression compared to DMSO treatment (Figure 6(e)). Prophylactic A33 treatment significantly lowered the C1QBP expression compared to prophylactic roflumilast treatment (*p* = 0.0426; Figure 6(e)). We did not observe significant differences in C1QBP expression between the different treatment groups in the lesion or the contralateral hemisphere (Figure 6(d) and (f)). This indicates that the C1QBP effect was situated in the penumbra. Additionally, we performed a series of double staining and revealed a co-localization with NeuN and identified neurons as the brain cells co-expressing C1QBP in these ischemic mouse brains (Figure 6(a)–(c)). Taken together, these immunofluorescent stainings confirm our findings following spatial proteomics analysis.

**Figure 6.**
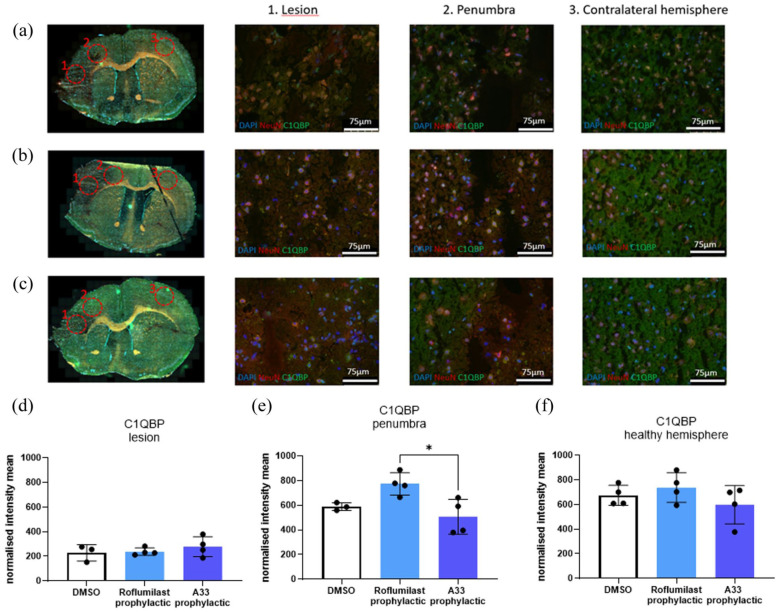
C1QBP immunofluorescent staining of mouse brain treated with PDE4 and PDE4B inhibition following experimental ischemic stroke. Mouse brains were stained using DAPI to stain the nucleus blue, as well as antibodies against C1QBP (green) and neuronal marker NeuN (red). Representative images of brain sections of mice treated with DMSO (a), roflumilast prophylactic (b), and A33 prophylactic (c), with more detailed images of the lesion (1), penumbra (2), and contralateral hemisphere (3). Quantification of the green fluorescent signal of the brain sections in the lesion (d), penumbra (e), and contralateral hemisphere (f). The mean fluorescent signal for each brain section was normalized against its negative control. Data are represented as mean ± SD. Analysis of the fluorescent signal was performed using Zeiss Lite 3.11 software. Statistical analysis Kruskal–Wallis was performed using GraphPad Prism software. *p < 0.05.

## Discussion

In this study, the effects of both pan-PDE4 and selective PDE4B or PDE4D inhibition were investigated in a stroke mouse model and validated in inflammatory neutrophils. Our results show that prophylactic, but not acute, pan-PDE4 and PDE4B inhibition reduce the lesion size and neutrophil infiltration following experimental ischemic stroke, whereas blockade of PDE4D was always ineffective. Additionally, both pan-PDE4 and PDE4B inhibition reduced neutrophil activation in human neutrophils in vitro.

Our in vivo results show that prophylactic pan-PDE4 and PDE4B inhibition reduce the lesion size while ameliorating neuroinflammation. This is in contrast to acute pan-PDE4 and PDE4B inhibition, which did not reveal a comparable effect in the dMCAO stroke mouse model. These results are partly in contrast to previous studies using a range of pan-PDE4 inhibitors, including rolipram, roflumilast, FCPR03, and FCPR16, that reported reduced lesion volumes and neuroinflammation when the pharmacological treatments started 2 h following stroke induction.^28,30–33,56^ This is consistent with our acute inhibition treatment set-up; however, our results did not show a significant effect following acute treatment. Interestingly, an important difference in our research compared to previous studies is the employed experimental stroke model. We used the dMCAO mouse model, inducing a permanent blockage of the middle cerebral artery, which translates to patients not receiving thrombectomy or thrombolysis. In contrast, all other studies used the transient middle cerebral artery occlusion (tMCAO) model in rats,^30–33,56^ or mice.^28^ The tMCAO model allows for reperfusion following ischemia, leading to additional inflammation and tissue damage, which is referred to as reperfusion injury, and is a representative model for patients receiving recanalization. This also contributes to the larger lesion volumes following tMCAO stroke compared to dMCAO stroke.^57–59^ The lack of reperfusion injury in our dMCAO model could potentially contribute to the lack of significant effect following acute pan-PDE4 inhibition compared to its effect following tMCAO. Another difference between our study and previous ones regards the dosage of the pan-PDE4 inhibitor. We treated mice with 3 or 10 mg/kg of roflumilast, while Kraft et al. employed 2 and 10 mg/kg of rolipram. A dose-dependent effect of rolipram was reported, with 10 mg/kg showing a significant reduction in lesion size 24 h following tMCAO in mice while 2 mg/kg only showed a trend towards reduced lesion size compared to the vehicle control.^28^ We did not observe such dose-dependent effect of roflumilast. Given this discrepancy between our findings and precious studies on the acute effects of pan-PDE4 inhibition, we considered the half-life of roflumilast, which is ~7 h in mice.^60^ This is encouraged by our observation that roflumilast is also no longer detectable in the brain after 24 h following subcutaneous injection.^61^ We therefore treated mice every 8 h with their respective treatment to improve plasma stability and brain concentrations up until 24 h post-stroke induction. This approach did not result in a dose-dependent effect of roflumilast on lesion size. Therefore, we concluded that treatment injections every 8 h did not hold an advantage over two treatment injections daily. Additionally, previous research reported improved functional outcome, decreased neurodegeneration in mice, and reduced neuroinflammation in rats following prolonged PDE4 inhibition after transient ischemia.^56,62^ However, we did not observe a significant effect on the lesion size following prolonged roflumilast and A33 treatment for 7 days after dMCAO stroke in mice. We also did not observe any effects of roflumilast, A33, or Gebr32a on functional recovery using the cylinder test up until 14 days post-stroke. Of note is that both Bonato et al. and Soares et al. studied the effects of pan-PDE4 inhibition for 21 days post-ischemia. Kraft et al. also reported a reduced number of infiltrated neutrophils after rolipram treatment compared to the vehicle control in the ischemic hemisphere 24 h following stroke.^28^ Previous research in a rat model for traumatic brain injury also reports reduced neuroinflammation with reduced numbers of infiltrated neutrophils and TNF-α concentrations following A33 treatment.^38,39^ Similarly, we observed a significant reduction in infiltrated neutrophils following prophylactic roflumilast and A33 treatment. However, acute treatment did not render this significant reduction in neutrophil infiltration 24 h following experimental ischemic stroke in our dMCAO model. A limitation of our study is the use of only male mice in the in vivo experiments. This can be explained by the higher stroke prevalence in males compared to females. Also, female sex hormones have been reported to exert a protective effect on lesion size and functional recovery following stroke.^63,64^ The Stroke Therapy Academic Industry Roundtable (STAIR) guidelines recommend that preclinical studies are performed in both male and female animals.^65^ However, due to the lack of effect on lesion size and neuroinflammation following acute PDE4 and PDE4B inhibition treatment in male mice, we did not repeat these experiments in female mice to also take reduction in the use of experimental animals into account. Even though acute pan-PDE4 and PDE4B inhibition did not seem to reduce the lesion size and neuroinflammation in our permanent stroke model, prophylactic treatment could potentially be translated to a subset of stroke patients, namely those at risk of recurrent stroke. For stroke patients, a 10% risk on stroke recurrence within 5 years of the first stroke exists.^18^ Even though advances have been made in secondary stroke prevention, a major part of this prevention treatment remains antithrombotic medication, which is not applicable for all stroke patients.^18–20^ Additionally, secondary stroke numbers remain relatively high throughout the years, highlighting the need for additional treatment strategies.^66,67^ Potentially, prophylactic PDE4B inhibition could become part of the recurrent stroke treatment in order to reduce the lesion volume and neuroinflammation following a secondary stroke. To the best of our knowledge, prophylactic pan-PDE4 and PDE4B inhibition have not been studied in experimental ischemic stroke. Results of pan-PDE4 inhibition using CHF6001II in an experimental model showing pulmonary inflammation, showed a significant reduction in infiltrated neutrophils following prophylactic treatment.^68^ However, more research into prophylactic inhibition treatment is needed, for example, into long-term effects of prophylactic PDE4 inhibition and into a combination therapy with other existing treatment options for ischemic stroke.

Our in vivo results on PDE4D inhibition using Gebr32a following dMCAO stroke in mice show no effect on lesion size and neuroinflammation. This is consistent with previous findings in a rat embolic stroke model, where PDE4D knock-out did not show an influence on lesion size compared to wild type controls.^69^ In contrast to our findings, research on PDE4D inhibition in other neurodegenerative diseases, including MS, TBI, spinal cord injury (SCI), and Alzheimer’s disease showed positive outcomes.^40,44,48,70,71^ More specifically, in both MS and SCI, PDE4D inhibition improved memory while stimulating remyelination.^40,44^ In Alzheimer’s disease, behavioral testing for memory deficits was improved following PDE4D inhibition.^48,71^ Also, a negative allosteric modulator of PDE4D ameliorated memory deficits in a rat model of TBI, 3 months following TBI induction.^70^ Also, in the studies on MS and SCI, mice were treated for a longer time period of 9 and 28 days, respectively.^40,44^ This could suggest that the PDE4D inhibition effect is situated more during the chronic phase of disease.

Because of the significant reduction of infiltrated neutrophils following prophylactic pan-PDE4 and PDE4B inhibition, we investigated the inhibition effect on stimulated human neutrophils in vitro. We reported significantly reduced ROS production by stimulated neutrophils following roflumilast and A33 treatment in a dose-dependent manner. Our results show that roflumilast optimally reduces neutrophil activation at 1 µM, whereas the optimal concentration for A33 was 0.1 µM. This is consistent with previous research on the effect of PDE4 inhibition on neutrophil activation.^72–75^ Roflumilast, apremilast, and several other PDE4 inhibitors were used to treat primary human neutrophils, and were all reported to reduce ROS production in a dose-dependent manner.^72–74^ Roflumilast was also reported to exert the strongest effect at 1 µM, which is in accordance with our results.^72^ Compound A, a PDE4B inhibitor, was reported to reduce neutrophil activation through reduced TNF-α production and a minor decreased chemotactic effect.^75^ These previous studies all isolated neutrophils out of human whole blood by means of a density gradient centrifugation, whereas we isolated neutrophils through immunomagnetic isolation.^72–75^ It has been reported that the means of neutrophil isolation exerts an influence on the cell’s activation. Namely, during the isolation process, neutrophils can become activated spontaneously. A density-gradient isolation was found to lead to higher spontaneous activation compared to immunomagnetic isolation when quantifying ROS production following isolation.^76^ This implies that in our ROS luminol assays neutrophils underwent less spontaneous activation prior to the added inflammatory stimulus because of the employed immunomagnetic isolation. As shown previously, PDE4B is the main isoform expressed in human neutrophils.^77,78^ Considering the effect of PDE4B inhibition on neutrophil activation, we were also interested in the PDE4B isoform expression in human neutrophils. It is already known that due to alternative promotors five different PDE4B isoforms are formed.^79^ These isoforms share their catalytic domain and C-terminal region but differ from each other in the N-terminal region and presence or absence of UCR1 and UCR2 regions.^37^ Very limited studies report about the PDE4B isoform expression. In 1999, a study reported that mainly PDE4B2 was found in human neutrophils following LPS stimulation. It is important to note that this study only mentions PDE4B1, PDE4B2, and PDE4B3 isoforms. Additionally, they employed 5′ RACE PCR to carry out the analysis, thus potentially missing some differences in UCR1 and UCR2 domains of the different isoforms.^77^ Another study identified the PDE4B isoforms in the mouse brain following LPS stimulation, and reported an upregulation in PDE4B2 expression in neutrophils found in the leptomeninges. Also, a reduction in PDE4B3 expression was observed in oligodendrocytes of the mouse brain.^80^ Our research results contradict these previous studies, since we mainly found PDE4B5 expression in human neutrophils, followed by PDE4B2 expression. Additionally, we analyzed non-stimulated neutrophils whereas previous research induced inflammatory activation. Considering a different tissue distribution of the PDE4B isoforms has been mentioned,^80^ more extensive qRT-PCR analyses with a larger sample size of PDE4B isoform expression throughout different cell types would be interesting also in a specific disease context. This could contribute to the incentive of isoform specific inhibition targeting a specific cell type in a pathological environment. We can conclude that mainly PDE4B5 and PDE4B2 are expressed in non-stimulated human neutrophils.

To gain a deeper understanding of the effect of prophylactic pan-PDE4 and PDE4B inhibition on proteins in the brain after stroke, we performed a spatial proteomics analysis on mouse brain samples. We identified C1QBP as an interesting protein following ischemic stroke. More specifically, we found that C1QBP was significantly downregulated in the CNS following prophylactic A33 treatment compared to the DMSO control. C1QBP is known to have multiple functions, including an extensive mitochondrial function and a contributing role in inflammation. Different cell types express the receptor for C1QBP on their cell surface, including B and T cells, monocytes, and macrophages; thereby contributing to the inflammatory function of C1QBP.^81,82^ It has been reported that the presence of C1QBP increased the inflammatory response by increasing apoptosis and ROS levels.^83^ Additionally, it also stimulates mitochondrial cell death which is a known contributor to disease pathogenesis.^84^ Furthermore, a decrease in levels of C1QBP in a mouse psoriasis model reduced inflammatory cell infiltration in these psoriatic skin lesions.^85^ Previous research has also investigated the role of C1QBP in molecular pathways. Connections between C1QBP and the p38 pathway and AKT/mTOR pathway have been suggested.^84,86^ Interestingly, C1q has also been investigated as a part of the complement system contributing to inflammation.^87^ We have demonstrated expression of C1QBP in neurons of the mouse brain. It is possible that C1QBP is released from damaged neurons and contributes to ischemic stroke pathogenesis by attracting neutrophils to the ischemic brain. It can be hypothesized that PDE4B inhibition indirectly suppresses neutrophil recruitment through a reduction in C1QBP expression. More research is needed into the effect of neuronal C1QBP in ischemic stroke.

A limitation of our study is the lack of patient validation using patient samples, such as post-mortem brain biopsies of ischemic stroke patients. However, post-mortem stroke brain biopsies are difficult to obtain. Also, many experimental factors need to be taken into account when working with post-mortem brain samples, such as age- and gender-matched controls, the type of stroke, site of the stroke lesion in the brain, and time and method of sample collection.^88,89^ However, the use of human stroke brain samples would render interesting research results and should be considered in future research.

Taken together, prophylactic pan-PDE4 and PDE4B inhibition reduce the lesion size and neutrophil infiltration following ischemic stroke in vivo. Additionally, spatial proteomics analysis reveals a reduction in C1QBP expression following prophylactic PDE4B inhibition. These results further corroborated in vitro, where both roflumilast (1 µM) and A33 (0.1 µM) reduce human neutrophil activation. Our research provides evidence for a potential role of prophylactic PDE4B inhibition in a subset of ischemic stroke patients, especially those at risk of recurrent stroke.

## Supplemental Material

sj-.jpg-6-jcb-10.1177_0271678X251386237 – Supplemental material for Prophylactic PDE4 and PDE4B inhibition reduce lesion size and neutrophil infiltration following ischemic stroke in male mice Supplemental material, sj-.jpg-6-jcb-10.1177_0271678X251386237 for Prophylactic PDE4 and PDE4B inhibition reduce lesion size and neutrophil infiltration following ischemic stroke in male mice by Laura Ponsaerts, Lotte Alders, Melissa Schepers, Hannelore Kemps, Elke Pirlet, Emily Willems, Mirre De Bondt, Ruben Jacobs, Chiara Brullo, Olga Bruno, Ernesto Fedele, Roberta Ricciarelli, Jos Prickaerts, Veerle Somers, Michiel Vandenbosch, Tim Vanmierlo and Annelies Bronckaers in Journal of Cerebral Blood Flow & Metabolism

sj-.jpg-7-jcb-10.1177_0271678X251386237 – Supplemental material for Prophylactic PDE4 and PDE4B inhibition reduce lesion size and neutrophil infiltration following ischemic stroke in male mice Supplemental material, sj-.jpg-7-jcb-10.1177_0271678X251386237 for Prophylactic PDE4 and PDE4B inhibition reduce lesion size and neutrophil infiltration following ischemic stroke in male mice by Laura Ponsaerts, Lotte Alders, Melissa Schepers, Hannelore Kemps, Elke Pirlet, Emily Willems, Mirre De Bondt, Ruben Jacobs, Chiara Brullo, Olga Bruno, Ernesto Fedele, Roberta Ricciarelli, Jos Prickaerts, Veerle Somers, Michiel Vandenbosch, Tim Vanmierlo and Annelies Bronckaers in Journal of Cerebral Blood Flow & Metabolism

sj-.jpg-8-jcb-10.1177_0271678X251386237 – Supplemental material for Prophylactic PDE4 and PDE4B inhibition reduce lesion size and neutrophil infiltration following ischemic stroke in male mice Supplemental material, sj-.jpg-8-jcb-10.1177_0271678X251386237 for Prophylactic PDE4 and PDE4B inhibition reduce lesion size and neutrophil infiltration following ischemic stroke in male mice by Laura Ponsaerts, Lotte Alders, Melissa Schepers, Hannelore Kemps, Elke Pirlet, Emily Willems, Mirre De Bondt, Ruben Jacobs, Chiara Brullo, Olga Bruno, Ernesto Fedele, Roberta Ricciarelli, Jos Prickaerts, Veerle Somers, Michiel Vandenbosch, Tim Vanmierlo and Annelies Bronckaers in Journal of Cerebral Blood Flow & Metabolism

sj-docx-1-jcb-10.1177_0271678X251386237 – Supplemental material for Prophylactic PDE4 and PDE4B inhibition reduce lesion size and neutrophil infiltration following ischemic stroke in male mice Supplemental material, sj-docx-1-jcb-10.1177_0271678X251386237 for Prophylactic PDE4 and PDE4B inhibition reduce lesion size and neutrophil infiltration following ischemic stroke in male mice by Laura Ponsaerts, Lotte Alders, Melissa Schepers, Hannelore Kemps, Elke Pirlet, Emily Willems, Mirre De Bondt, Ruben Jacobs, Chiara Brullo, Olga Bruno, Ernesto Fedele, Roberta Ricciarelli, Jos Prickaerts, Veerle Somers, Michiel Vandenbosch, Tim Vanmierlo and Annelies Bronckaers in Journal of Cerebral Blood Flow & Metabolism

sj-tif-2-jcb-10.1177_0271678X251386237 – Supplemental material for Prophylactic PDE4 and PDE4B inhibition reduce lesion size and neutrophil infiltration following ischemic stroke in male mice Supplemental material, sj-tif-2-jcb-10.1177_0271678X251386237 for Prophylactic PDE4 and PDE4B inhibition reduce lesion size and neutrophil infiltration following ischemic stroke in male mice by Laura Ponsaerts, Lotte Alders, Melissa Schepers, Hannelore Kemps, Elke Pirlet, Emily Willems, Mirre De Bondt, Ruben Jacobs, Chiara Brullo, Olga Bruno, Ernesto Fedele, Roberta Ricciarelli, Jos Prickaerts, Veerle Somers, Michiel Vandenbosch, Tim Vanmierlo and Annelies Bronckaers in Journal of Cerebral Blood Flow & Metabolism

sj-tif-3-jcb-10.1177_0271678X251386237 – Supplemental material for Prophylactic PDE4 and PDE4B inhibition reduce lesion size and neutrophil infiltration following ischemic stroke in male mice Supplemental material, sj-tif-3-jcb-10.1177_0271678X251386237 for Prophylactic PDE4 and PDE4B inhibition reduce lesion size and neutrophil infiltration following ischemic stroke in male mice by Laura Ponsaerts, Lotte Alders, Melissa Schepers, Hannelore Kemps, Elke Pirlet, Emily Willems, Mirre De Bondt, Ruben Jacobs, Chiara Brullo, Olga Bruno, Ernesto Fedele, Roberta Ricciarelli, Jos Prickaerts, Veerle Somers, Michiel Vandenbosch, Tim Vanmierlo and Annelies Bronckaers in Journal of Cerebral Blood Flow & Metabolism

sj-tif-4-jcb-10.1177_0271678X251386237 – Supplemental material for Prophylactic PDE4 and PDE4B inhibition reduce lesion size and neutrophil infiltration following ischemic stroke in male mice Supplemental material, sj-tif-4-jcb-10.1177_0271678X251386237 for Prophylactic PDE4 and PDE4B inhibition reduce lesion size and neutrophil infiltration following ischemic stroke in male mice by Laura Ponsaerts, Lotte Alders, Melissa Schepers, Hannelore Kemps, Elke Pirlet, Emily Willems, Mirre De Bondt, Ruben Jacobs, Chiara Brullo, Olga Bruno, Ernesto Fedele, Roberta Ricciarelli, Jos Prickaerts, Veerle Somers, Michiel Vandenbosch, Tim Vanmierlo and Annelies Bronckaers in Journal of Cerebral Blood Flow & Metabolism

sj-tif-5-jcb-10.1177_0271678X251386237 – Supplemental material for Prophylactic PDE4 and PDE4B inhibition reduce lesion size and neutrophil infiltration following ischemic stroke in male mice Supplemental material, sj-tif-5-jcb-10.1177_0271678X251386237 for Prophylactic PDE4 and PDE4B inhibition reduce lesion size and neutrophil infiltration following ischemic stroke in male mice by Laura Ponsaerts, Lotte Alders, Melissa Schepers, Hannelore Kemps, Elke Pirlet, Emily Willems, Mirre De Bondt, Ruben Jacobs, Chiara Brullo, Olga Bruno, Ernesto Fedele, Roberta Ricciarelli, Jos Prickaerts, Veerle Somers, Michiel Vandenbosch, Tim Vanmierlo and Annelies Bronckaers in Journal of Cerebral Blood Flow & Metabolism
