# Supplementary material for: Prophylactic PDE4 and PDE4B inhibition reduce lesion size and neutrophil infiltration following ischemic stroke in male mice
Source: J Cereb Blood Flow Metab. 2025 Nov 3;46(4):896–912. doi: 10.1177/0271678X251386237 (PMC12586380; doi:10.1177/0271678X251386237)
Supplement: Supplementary file 1 [file sj-docx-1-jcb-10.1177_0271678X251386237.docx]

Figure legends of Supplemental figures

**Supplementary Figure 1** Acute PDE4 inhibition treatment does not influence lesion size following increased dosage or prolonged exposure. (**A**) Timeline of the acute treatment experiment as assessed 24 hours after ischemic stroke. Mice received their first subcutaneous treatment injection 2 hours following dMCAO surgery, and received a second treatment injection 22 hours post-stroke induction. Treatment groups included: DMSO (0.3%, vehicle control), roflumilast (3 or 10mg/kg, PDE4 inhibitor). 24 hours following dMCAO stroke, brains were isolated for TTC staining. Created with Biorender.com (**B**) Representative TTC images. (**C**) Quantification of lesion size (TTC images). (n=8-10/group) (**D**) Timeline of the acute treatment experiment with injections every 8 hours. Mice received a first subcutaneous treatment injection 30 minutes following dMCAO surgery, and received following treatment injections every 8 hours up until 24 hours post-stroke. Treatment groups included: DMSO (0.3%), roflumilast (3 or 10mg/kg). Lesion size was assessed using TTC staining 24 hours after dMCAO surgery. Created with BioRender.com (**E**) Representative TTC images. (**F**) Quantification of lesion size. (n=9-10/group) (**G**) Timeline of the acute prolonged treatment experiment. Mice received a first subcutaneous injection 2 hours following dMCAO stroke surgery. The treatment injections were repeated 2x/day for a period of 7 days, after which brains were isolated for TTC staining. Treatment groups included: DMSO (0.1%), roflumilast (3mg/kg), A33 (3mg/kg, PDE4B inhibitor), and Gebr32a (3mg/kg, PDE4D inhibitor). Created with BioRender.com (**H**) Representative TTC images. (**I**) Quantification of lesion size. (n=10/group) Data are represented as mean ± SD. Statistical analysis included one-way ANOVA with a Tukey correction for multiple comparisons.

**Supplementary Figure 2** Acute PDE4, PDE4B, and PDE4D inhibition do not influence functional recovery following dMCAO stroke. (**A**) Timeline of the experiment. Mice received their subcutaneous injections 2 hours post-stroke induction. Treatment injections were repeated 2x/day for a period of 14 days following dMCAO surgery. Behavioral assessment was performed using the cylinder test at baseline (1 day prior to stroke induction), and at days 1, 3, 7, and 14 following ischemic stroke. Treatment groups included: DMSO (0.1%, vehicle control), roflumilast (3mg/kg, PDE4 inhibitor), A33 (3mg/kg, PDE4B inhibitor), and Gebr32a (3mg/kg, PDE4D inhibitor). (B) Representation of the cylinder test. A mouse is placed inside a see-through cylinder, and is allowed to explore the walls of the cylinder. Mice will explore the cylinder by standing on their rear legs and touching the cylinder wall using their front paw. The amount of ipsilateral and contralateral paw touches are quantified, and the %impaired forelimb use is calculated by dividing the number of contralateral paw touches by the toal number of paw touches and multiplying this by 100. Then normalization is done by dividing the %impaired forelimb use by the %impaired forelimb use at baseline. Quantification of the impaired forelimb use at days 1 (**C**), 3 (**D**), 7 (**E**), and 14 (**F**) post-stroke. Data are represented as mean ± SD. Statistical analysis included one-way ANOVA analysis with a Tukey correction for multiple comparisons. Created with Biorender.com

**Supplementary Figure 3** Flow cytometry gating strategy. Doublets were removed based on the forward scatter (FSC) and side scatter (SSC). Next, living cells were selected based on a Zombie live/dead staining after which leukocytes (CD45+ cells) were selected. Neutrophils were identified as cells positive for CD45, CD11b, and Ly6G^high^. Macrophages were identified as cells positive for CD45, CD11b, Ly6C^high^, and arginase. Monocytes were identified as cells positive for CD45, CD11c, Ly6G^low^. Flow cytometry analysis was performed using a BD LSRF Fortessa flow cytometer.

**Supplementary Figure 4** Annotation of the region of interest for spatial proteomics. Cryosections of mouse brains of 10µm thick were made and stained using cresylviolet 0.1% to visualize the stroke lesion after dMCAO stroke induction. The region of interest, namely the penumbra was annotated (yellow figure on the brain scan) using QuPath-0.5.1 following digitalization of the brain sections using the axioscan Z.
